# Efficacy and safety of pateclizumab (anti-lymphotoxin-α) compared to adalimumab in rheumatoid arthritis: a head-to-head phase 2 randomized controlled study (The ALTARA Study)

**DOI:** 10.1186/s13075-014-0467-3

**Published:** 2014-10-30

**Authors:** William P Kennedy, J Abraham Simon, Carolyn Offutt, Priscilla Horn, Ann Herman, Michael J Townsend, Meina T Tang, Jane L Grogan, Frank Hsieh, John C Davis

**Affiliations:** Genentech Research and Early Development, 1 DNA Way, South San Francisco, CA 94080 USA; Unidad de Investigación Médica, UMAE-IMSS, C. 34 No 439 x 41, Colonia Industrial, CP 97150, Mérida, Yucatán Mexico

## Abstract

**Introduction:**

Tumor necrosis factor (TNF) and, possibly, lymphotoxin alpha (LTα) signaling contribute to inflammation and rheumatoid arthritis (RA) pathogenesis. Pateclizumab (anti-lymphotoxin- alpha; MLTA3698A) is a humanized monoclonal antibody that blocks and depletes anti-LTα. This phase 2, randomized, head-to-head, active- and placebo-controlled trial examined the safety and efficacy of pateclizumab compared to adalimumab in RA patients with an inadequate response to disease-modifying antirheumatic drugs (DMARD-IR).

**Methods:**

Patients (*n* = 214) with active RA (≥6 swollen and tender joints, C-reactive protein ≥10 mg/L) on oral DMARDs were randomized (2:2:1) to receive pateclizumab 360 mg, adalimumab 40 mg, or placebo subcutaneously every 2 weeks. The primary endpoint, 4-variable, 28-joint disease activity score erythrocyte sedimentation rate (DAS28(4)-ESR) response, was evaluated at 12 weeks using an analysis of covariance (ANCOVA) model with adjustments for concomitant DMARD use and geographic region. Secondary efficacy endpoints included American College of Rheumatology (ACR) 20, ACR50, and ACR70 responses at Day 85. Pharmacokinetics, pharmacodynamics, and immunogenicity of pateclizumab were assessed.

**Results:**

Pateclizumab reduced the DAS28(4)-ESR response (−1.89) at 12 weeks, however, this did not reach statistical significance compared to placebo (−1.54), while adalimumab (−2.52) differed significantly from both placebo and pateclizumab. Pateclizumab 12-week ACR20, ACR50 and ACR70 response rates (64%, 33%, and 14%) suggested clinical activity but were not statistically significant compared to placebo rates (46%, 24%, and 8%, respectively). CXCL13 serum levels decreased significantly following pateclizumab and adalimumab administration, demonstrating pharmacological target engagement by both drugs. Overall, adverse events (AEs) were comparable among all cohorts. Infections were the most common AE, occurring with comparable frequency in all groups. Serious AEs occurred in 0% of pateclizumab, 5.9% of adalimumab, and 2.3% of placebo patients, with serious infection in 2.3% of adalimumab patients and none in pateclizumab and placebo patients.

**Conclusions:**

Pateclizumab had a good safety profile in patients inadequately responsive to DMARDs, but no statistically significant improvement in RA signs and symptoms after 12 weeks of treatment. Adalimumab demonstrated efficacy and safety comparable to published results in this head-to-head comparison in DMARD-IR RA patients.

**Trial registration:**

ClinicalTrials.gov NCT01225393, Registered 18 October 2010.

## Introduction

The risk of disease progression in rheumatoid arthritis (RA) occurs in patients who do not respond adequately to available treatment options [1]. Despite the advances with biological agents for the treatment of RA, there remains a significant unmet need for those who do not respond to these agents.

Synovitis in RA develops as a result of infiltration of innate and adaptive immune cells causing a significant inflammatory response and cytokine release, including, but not limited to tumor necrosis factor alpha (TNF-α). During chronic inflammation these cellular infiltrates organize into *de novo* lymphoid aggregates. These ectopic lymphoid aggregates are instigated and maintained by lymphotoxin (LT)-mediated pathways. Lymphotoxin alpha (LTα) and beta (LTβ) are two related TNF superfamily (TNFSF) members produced predominately by activated cells of the innate and adaptive immune response. LTα exists as a secreted homotrimeric molecule (LTα_3_) that signals via TNF receptor 1 (TNFR1) and TNFR2 to promote inflammation; whereas LTα complexed with LTβ (LTα1β2), on the surface of T and B cells, signals through the LTβ receptor (LTβR) [2-4]. Lymphoid aggregates in the synovium of RA patients are associated with LTβ expression, and production of B cell attractant chemokine CXCL13 [5,6]. B cells in ectopic lymphoid tissue samples from the lungs of RA patients with pulmonary complications produce rheumatoid factor (RF) and autoantibodies to citrullinated proteins [7]. Moreover, the CD4 T helper (Th) subsets Th1 and Th17, which have been implicated in RA and other autoimmune diseases, express high levels of surface LTα1β2, and depletion of these cells with a murine anti-LTα-depleting antibody has demonstrated therapeutic efficacy in preclinical murine models of RA [4]. In comparison, mice treated with the LTβR decoy fusion protein, were only afforded efficacy when treated preventively, consistent with the failure of LTβR immunoglobulin (LTβR-Ig) to meet clinical endpoints in clinical trials in RA patients [8,9].

Given the role lymphotoxin, TNF-α, and Th cells may play in inflammation and RA pathogenesis, and the additional role soluble LTα3 may play in disease pathogenesis [10-15], we generated a fully humanized blocking and depleting anti-LTα monoclonal antibody (MLTA3698A, pateclizumab) that blocks LTα_3_-TNFR interactions, LTα_1_β_2_-LTβR interactions, specifically depletes LTα1β2-expressing lymphocytes [4]. In a phase I study in patients with active RA, pateclizumab was well tolerated and provided preliminary evidence of clinical activity [16]. Here, we report on the ALTARA (Anti-LTa Rheumatoid Arthritis) study - a phase 2, randomized, head-to-head, active- and placebo-controlled trial of the safety and efficacy of pateclizumab compared to adalimumab in RA patients who had an inadequate response to disease-modifying antirheumatic drugs (DMARD-IR).

## Methods

### Study design

The ALTARA study was conducted in 47 centers in 10 countries, including sites in the United States, Europe, and Latin America. The study was conducted in compliance with the Declaration of Helsinki and the International Conference on Harmonization Good Clinical Practice Guidelines. It was approved by the Comités de Ética e Investigación en Salud del Centro de Especialidades Médicas del Sureste S.A. de C.V. (Unidad de Investigacion Biomedica del Cem). All patients provided written informed consent before performance of any study procedures.

### Dose selection

Pateclizumab was administered as subcutaneous (SC) injections, 360 mg every other week. This study regimen was selected based on the following considerations: 1) the total exposure was, on average, 60% higher than the 3 mg/kg biweekly SC doses evaluated in the pateclizumab phase I study; 2) this regimen was expected to result in a maximal pharmacological effect as suggested by plateaued reductions in serum CXCL13 level in all dose groups at 1-mg/kg or higher doses; and 3) this regimen has 2.2- to 4.5-fold exposure safety coverage by the highest exposure level assessed in the phase I study [16]. An SC biweekly dose of 360 mg pateclizumab was expected to maximize the chance of demonstrating clinical activity while having sufficient safety coverage. Adalimumab was administered as 40-mg SC injections every other week.

### Entry criteria

Eligible patients were ≥18 years of age, had a diagnosis of RA for ≥6 months (meeting the American College of Rheumatology (ACR) 1987 revised criteria for RA [17]), and had active disease, defined as ≥6 tender/painful joints (68-joint count) and ≥6 swollen joints (66-joint count) and a C-reactive protein (CRP) level ≥10 mg/L at the screening and baseline visits. Additional key inclusion criteria included failure of at least one DMARD, including methotrexate or leflunomide, with current regimen stable for ≥3 months.

Key exclusion criteria included the following: previous biologic therapy for RA; evidence of hematopoietic disorders at screening or within 3 months; creatinine >1.5 × upper limit of normal (ULN), aspartate aminotransferase (AST) or alanine aminotransferase (ALT) levels 1.5 × ULN; positive QuantiFeron [18] results for latent and/or active *Mycobacterium tuberculosis*; clinically significant infection(s); and a history of malignancy, with the exception of adequately treated nonmetastatic basal cell or squamous cell cancer of the skin or cervical carcinoma *in situ*.

### Randomization and study treatment

This was a phase 2, randomized, double-blind, active- and placebo-controlled, parallel-group study. Patients were randomized 2:2:1 to receive one of the following treatments: pateclizumab 360 mg, adalimumab 40 mg, or placebo. The study drug was administered SC once every other week for 10 weeks (baseline (Week 0) and Weeks 2, 4, 6, 8, and 10). We were unable to construct a placebo syringe corresponding to the manufactured syringe supplied with adalimumab. As a result, unblinded medical personnel administered the allocated treatment while maintaining a strict blind for the patients and medical personnel associated with safety, joint, and efficacy evaluations.

### Concomitant medications

Patients continued on a stable background regimen of RA therapy, which included antimalarial agents, nonsteroidal anti-inflammatory drugs, acetaminophen, and/or oral corticosteroids (<10 mg prednisone or equivalent/day). Patients continued on either methotrexate or leflunomide at protocol-defined stable doses throughout the study. Intra-articular corticosteroids were prohibited in the 6 weeks prior to screening; they were allowed in one joint during the study, but were discouraged between Weeks 10 and 12. For all injected joints, the treated joint was censored from the efficacy analysis from the time of injection up to a maximum of 12 weeks.

### Study assessments

#### Efficacy measures

The primary objective was to compare the efficacy of pateclizumab to that of adalimumab by assessing the change from baseline in the 4-variable, 28-joint disease activity score erythrocyte sedimentation rate (DAS-ESR(4)) on Day 85 (Week 12).

Secondary objectives included the durability of the pateclizumab treatment response, safety of the treatment over 24 weeks, and the efficacy of adalimumab over 12 weeks. The secondary efficacy endpoints analyzed throughout the study included the following: the ACR20, ACR50, and ACR70 response rates, and the proportions of patients achieving disease remission, according to the 3-variable disease activity score in 28 joints (DAS28) [19] using the CRP (DAS28-CRP), or DAS28 score of <2.6, and the least squares mean change in components of the ACR core set of disease activity measures, including the Health Assessment Questionnaire disability index (HAQ-DI) [20], the 36-item Short Form (SF-36) Health Survey [21], and the patient’s assessment of pain and patient’s and physician’s global assessments of disease activity on 0 to 100-mm visual analog scales.

#### Safety measures

Safety was evaluated by nature, severity, and drug relation of adverse events (AEs), graded according to National Cancer Institute Common Toxicity Criteria for Adverse Events (NCI-CTCAE v3.0) and reported by incidence. Vital signs, physical examination findings, concomitant medications, and laboratory and pregnancy test results were collected every 2 weeks during the study. Incidence of anti-therapeutic antibodies (ATA) to pateclizumab in serum was evaluated using a bridging enzyme-linked immunosorbent assay (ELISA) on Days 57, 85, and 155 or early termination.

### Pharmacokinetics and immunogenicity assessment

For pharmacokinetic (PK) assessment, serum samples were obtained at predose Day 1; at 4 hours and at 3 and 6 days after the first dose; predose on Days 15, 29, 57, and 71; 3 and 14 days after final dose, and during drug washout for up to 12 weeks. The PK samples were analyzed using a validated ELISA with a lower limit of quantification of 100 ng/mL pateclizumab.

For ATA assessment, serum samples were obtained at predose and multiple postdose time points (Days 57, 85, 155, or early termination) and assessed using a bridging ELISA [16]. The relative sensitivity of the assay was estimated to be 50 ng/mL using an anti-pateclizumab complementarity-determining region (CDR) antibody. The assay was optimized to minimize interference by RF and by pateclizumab, and could detect 500 ng/mL of the anti-CDR antibody in the presence of 50 μg/mL of pateclizumab.

### Pharmacodynamic biomarker assessment

CXCL13 was measured as a pharmacodynamic biomarker, as identified in the phase 1 study [16,22]. Serum samples were obtained predose on Day 1 and postdose on Days 15, 29, 57, 71, 74, 85, 113, 141, and 155. A bridging electrochemiluminescence assay (ECLA) was validated to quantify CXCL13 in RA serum. Briefly, samples were incubated with two anti-CXCL13 antibody reagents, one labeled with biotin. Samples were captured using streptavidin, and measured using standard immunoassay methods (minimum quantifiable concentration, 20 pg/mL). Patients without detectable CXCL13 at baseline (n = 14) or with changes >1,000% of baseline (n = 2) were excluded from analysis.

### Statistical methods

All efficacy analyses were performed using the modified intent-to-treat (mITT) populations; all safety analyses were performed using the as-treated population. The primary efficacy endpoint of change of DAS28(4)-ESR at Day 85 was assessed using an analysis of covariance (ANCOVA) model with adjustment of concomitant DMARD use, geographic region, and baseline value. The secondary efficacy endpoints include ACR20, ACR50, and ACR70 responses at Day 85. Any missing ACR score components were imputed with the last-observation-carried-forward (LOCF) method. The *P* values for comparing treatment differences in ACR20/50/70 responses were based on the Cochran-Mantel-Haenszel test with adjustment for stratification factors of geographic region and concomitant DMARD use. The *P* values were not corrected for multiple comparisons.

## Results

### Patient disposition and baseline characteristics

Overall, 214 patients were randomized to receive study treatment. Of these, 180 received study treatment and completed the study for efficacy evaluations (mITT). The 34 patients withdrawn prior to the completion of the study included 13 patients treated with pateclizumab, 11 treated with adalimumab, and 10 treated with placebo. Reasons for early withdrawal included AE (n = 7), patient decision (n = 17), sponsor decision (n = 8), and loss to follow-up from the pateclizumab cohort (n = 2). An additional 11 patients did not meet eligibility criteria. Thus, of the 214 patients, 170 who received a study treatment were included in efficacy and safety evaluations.

Randomization achieved treatment groups that were well balanced for baseline demographic and clinical characteristics (Table 1). The majority of patients were white and female; the mean age was 50.1 years. The mean duration of RA disease was 7.2 to 9.3 years, and 84 to 87% of patients were on methotrexate at randomization.

**Table 1:** **Demographic and baseline characteristics**

	**Pateclizumab**	**ADA**	**Placebo**	**All patients**
	**(n = 85)**	**(n = 85)**	**(n = 44)**	**(n = 214)**
Age (yr)				
Mean (SD)	50.2 (13.1)	50.6 (13.3)	48.8 (14.0)	50.1 (13.3)
Median	51.0	52.0	48.5	51.0
Range (min, max)	18-75	20-73	23-75	18-75
Age group				
<65 yr (%)	72 (84.7%)	69 (81.2%)	36 (81.8%)	177 (82.7%)
≥65 yr (%)	13 (15.3%)	16 (18.8%)	8 (18.2%)	37 (17.3%)
Sex				
Female	78 (91.8%)	68 (80.0%)	37 (84.1%)	183 (85.5%)
Male	7 (8.2%)	17 (20.0%)	7 (15.9%)	31 (14.5%)
Race				
White	53 (62.4%)	48 (56.5%)	29 (65.9%)	130 (60.7%)
Black	1 (1.2%)	0 (0.0%)	0 (0.0%)	1 (0.5%)
American Indian or Alaska Native	1 (1.2%)	3 (3.5%)	0 (0.0%)	4 (1.9%)
Asian	(0.0%)	0 (0.0%)	0 (0.0%)	0 (0.0%)
Native Hawaiian or other Pacific Islander	(0.0%)	0 (0.0%)	0 (0.0%)	0 (0.0%)
Not available	30 (35.3%)	34 (40.0%)	15 (34.1%)	79 (36.9%)
Region				
US and Western Europe	12 (14.1%)	12 (14.1%)	6 (13.6%)	30 (14.0%)
Latin America and Eastern Europe	73 (85.9%)	73 (85.9%)	38 (86.4%)	184 (86.0%)
BMI (kg/m^2^)				
Mean (SD)	27.114 (5.075)	27.309 (4.194)	26.903 (5.547)	27.148 (4.830)
Weight (kg)				
Mean (SD)	68.66 (14.88)	69.52 (12.75)	69.15 (22.61)	69.11 (15.96)

ADA, adalimumab; BMI, body mass index; min, max, minimum, maximum; SD, standard deviation.

### Primary efficacy endpoint: DAS(4)-ESR at Week 12

Pateclizumab had minimal clinical activity by DAS28(4)-ESR but this did not reach statistical significance compared to placebo at Week 12. In contrast, adalimumab demonstrated a robust treatment effect at Week 12. The mean changes from baseline in DAS28(4)-ESR at Day 85 were −1.89, −2.52, and −1.54 for the pateclizumab, adalimumab, and placebo groups, respectively (Table 2). The differences between adalimumab and the other two treatment groups in DAS28(4)-ESR mean change from baseline were statistically significant (*P* <0.01) whereas the difference between the pateclizumab and placebo groups was not (*P* >0.05).

**Table 2:** **Primary (DAS28-ESR) and key secondary endpoints at day 85**

	**Pateclizumab**	**ADA**	**Placebo**
	**(n = 84)**	**(n = 84)**	**(n = 43)**
DAS28(4)-ESR score at baseline			
Mean (SD)	6.95 (0.89)	6.84 (0.90)	6.80 (0.74)
DAS28(4)-ESR score at day 85			
Mean (SD)	5.06 (1.52)	4.31 (1.46)	5.26 (1.44)
DAS28(4)-ESR score change from baseline			
Mean (SD)	–1.89 (1.38)	–2.52 (1.43)	–1.54 (1.34)
*P* value of difference of change score^a^			
Versus placebo	0.5172	0.0004	–
Versus ADA	0.0003	–	–
ACR20 response			
n (%)	50 (64.1%)	58 (77.3%)	17 (45.9%)
ACR50 response			
n (%)	26 (33.3%)	43 (57.3%)	9 (24.3%)
ACR70 response			
n (%)	11 (14.1%)	26 (34.7%)	3 (8.1%)
ACR components, mean (*P* value vs. placebo^b^)			
Swollen joint count	–8.9 (0.04)	–10.4 (<0.01)	–6.1
Tender joint count	–13.3 (0.10)	–16.3 (<0.01)	–9.8
Patient’s global VAS (mm)	–28.8 (0.27)	–37.0 (<0.01)	–24.0
Physician’s global VAS (mm)	–33.8 (<0.01)	–34.1 (<0.01)	–23.2
Patient’s pain VAS	–26.7 (0.24)	–33.5 (<0.01)	–21.6
CRP	–0.5 (0.08)	–1.2 (<0.01)	0.3
ESR	–11.9 (0.47)	–22.9 (0.04)	–14.7
HAQ-DI	–0.5 (0.09)	–0.8 (<0.01)	–0.3

^a^ *P* value from analysis of covariance; ^b^ *P* value for least squares mean change from baseline compared with placebo. ACR, American College of Rheumatology; ADA, adalimumab; CRP, C-reactive protein; DAS28(4), 4-variable, 28-joint disease activity score; ESR, erythrocyte sedimentation rate; HAQ-DI, Health Assessment Questionnaire disability index; SD, standard deviation; VAS, visual analog scale (0 to 100).

### Secondary efficacy endpoints

The ACR50 response was achieved at Day 85 in 26 (33.3%), 43 (57.3%), and 9 (24.3%) patients from the pateclizumab, adalimumab, and placebo groups, respectively (Table 2). The differences between adalimumab and the two other treatment groups in ACR50 rates were statistically significant (*P* <0.01), but the difference between pateclizumab and placebo was not (*P* >0.05). Pateclizumab failed to reach statistical significance for other key secondary efficacy endpoints, including ACR20 and ACR70. Pateclizumab was significantly different from placebo for two components of the composite ACR score: swollen joint count (*P* <0.04) and physician’s global visual analog scale (VAS) (*P* <0.01). Adalimumab significantly differed from placebo in each of the ACR components. In addition, SF-36 individual and component summary scores also showed a statistically significant difference between adalimumab and pateclizumab but not between pateclizumab and placebo.

### Safety and tolerability

Pateclizumab was generally safe and well tolerated in this study. No deaths occurred. Six patients had serious AEs (SAEs): five patients who received adalimumab and one patient who received placebo. Of the six SAEs, two (pulmonary tuberculosis (TB) and lymphopenia, both in the adalimumab group) were judged by the investigator to be related to the study drug. No patients were withdrawn from treatment due to an SAE.

AEs in the pateclizumab cohort were comparable in frequency to the placebo rates (Table 3). In the vast majority of patients, AEs were Grade 1 or 2 in severity; however, six patients in the pateclizumab group, eight in the adalimumab group, and three in the placebo group experienced AEs of Grade 3 or higher. There was one patient from Peru in the adalimumab-treatment group who had a negative interferon-gamma release assay screening result for latent and/or active TB who presented at randomization on study day 1 with a cough and subsequently developed an active TB infection.

**Table 3:** **Summary of number and percentage of subjects with treatment-emergent adverse events**

	**Pateclizumab**	**ADA**	**Placebo**
	**(n = 86)**	**(n = 85)**	**(n = 43)**
Any AE	50 (58%)	65 (76%)	31 (72%)
Any SAE	0 (0%)	5 (6%)	1 (2%)
Any AE Grade ≥3	6 (7%)	8 (9%)	3 (7%)
Any drug-related AE	20 (23%)	26 (31%)	10 (23%)
Any AE within 24 hr of dosing	7 (8%)	27 (32%)	6 (14%)
Any SAE within 24 hr of dosing	0 (0%)	0 (0%)	0 (0%)
Any drug-related AE within 24 hr of dosing	4 (5%)	12 (14%)	5 (12%)
Any infection AE	25 (29%)	32 (38%)	19 (44%)
Any infection SAE	0 (0%)	2 (2%)	0 (0%)
Any infection AE Grade 3 or higher	0 (0%)	1 (1%)	0 (0%)
Any AE leading to discontinuation of study drug	2 (2%)	6 (7%)	1 (2%)
Any death	0 (0%)	0 (0%)	0 (0%)

ADA, adalimumab; AE, adverse event; SAE, serious adverse event.

The most frequent (≥3%) treatment-emergent AEs are listed in Table 4. From baseline to Week 12, the most frequently reported AEs were pharyngitis, headache, urinary tract infection, hypertension, nasopharyngitis anemia, diarrhea, elevated ALT level, rheumatoid arthritis, and gastroenteritis. No clinically significant changes in laboratory parameters or vital signs were observed in the pateclizumab cohort.

**Table 4:** **Summary of treatment-emergent adverse events that occurred ≥3% in any treatment group**

	**Pateclizumab**	**ADA**	**Placebo**
	**(n = 86)**	**(n = 85)**	**(n = 43)**
Pharyngitis	7 (8.1%)	7 (8.2%)	4 (9.3%)
Headache	3 (3.5%)	11 (12.9%)	2 (4.7%)
Urinary tract infection	7 (8.1%)	7 (8.2%)	2 (4.7%)
Hypertension	5 (5.8%)	5 (5.9%)	1 (2.3%)
Nasopharyngitis	4 (4.7%)	4 (4.7%)	3 (7.0%)
Anemia	5 (5.8%)	3 (3.5%)	2 (4.7%)
Diarrhea	2 (2.3%)	5 (5.9%)	3 (7.0%)
Alanine aminotransferase increased	2 (2.3%)	3 (3.5%)	4 (9.3%)
Rheumatoid arthritis	3 (3.5%)	3 (3.5%)	3 (7.0%)
Gastroenteritis	3 (3.5%)	3 (3.5%)	1 (2.3%)

ADA, adalimumab.

### Pharmacokinetics, pharmacodynamics, and immunogenicity

**Figure 1:**
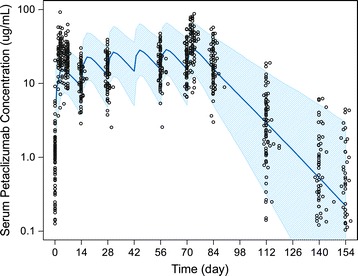
**Observed versus population PK model predicted pateclizumab serum concentration-time profiles.** Dose was given on study days 0, 14, 24, 42, 56, and 70. The empty symbols represent the observed individual serum concentrations at planned sampling times. The solid line represents the population PK model (built based on phase I PK data) predicted median serum concentration-time profile. The shaded band represents model predicted 90% confidence interval of the concentration-time profile for the study population. PK, pharmacokinetic.

Following six biweekly SC doses of 360 mg pateclizumab, the maximum mean ± standard deviation (SD) observed serum concentration was 41.1 ± 18.4 μg/mL, and occurred on Day 74 (3 days following the sixth dose). The observed steady-state average trough concentration (C_trough,ss_) was 21.3 ± 12.0 μg/mL. The mean trough concentration ratio of Day 85 (14 days after the last dose) to Day 15 (14 days after the first dose) was 1.79, indicating a mild exposure accumulation. The observed PK profile was well aligned with the profile predicted by a population PK model built using the PK data from the pateclizumab phase I study (Figure 1).

**Figure 2:**
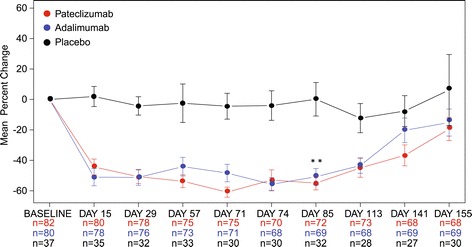
**B cell chemoattractant CXCL13 was decreased after treatment with pateclizumab.** Mean ± SEM levels of CXCL13, a B cell chemoattractant, are presented over time after treatment with adalimumab, pateclizumab, or placebo. CXCL13 was decreased following treatment with pateclizumab, demonstrating pharmacological engagement of the LTα pathway, as observed in the phase 1 trial of pateclizumab [12]. Adalimumab also modulated CXCL13 levels to a similar degree. Significance was assessed at the efficacy endpoint (Day 85); see Methods. *P <0.05 vs. placebo. LTα, lymphotoxin alpha; SEM, standard error of the mean.

Pre- and postdose levels of serum CXCL13 were evaluated as a biomarker for target modulation. The three treatment groups did not significantly differ in mean (± SD) CXCL13 baseline levels (pg/mL). Serum CXCL13 levels decreased rapidly and significantly in patients treated with pateclizumab (Figure 2), demonstrating evidence of an on-target pharmacological effect (*P* <0.01, pateclizumab vs. placebo, Day 85). Adalimumab also decreased CXCL13 levels significantly (*P* <0.01, adalimumab vs. placebo, Day 85), as expected [16]. Serum CXCL13 levels returned to predose levels for both treatment cohorts during the safety follow-up. As a comparison, such a treatment pharmacological effect was not observed in the placebo-treated group.

Among the 85 patients who were treated with pateclizumab, four patients were positive for ATA, and all positive ATA signals were detected at a single time point on Day 155, approximately 3 months after the final pateclizumab dose.

## Discussion

To our knowledge, ALTARA is the first phase 2 study directly comparing the safety and efficacy of an investigational agent, pateclizumab, with an approved anti-TNF product for the treatment of DMARD-IR patients on background DMARDs. Efficacy was directly compared in a head-to-head study for 3 months of treatment, which is considered a sufficient duration of treatment for such comparisons [23,24]. Since adalimumab combined with the standard-of-care methotrexate is one of the most common therapeutic choices for patients with RA and an inadequate response to DMARDs, it is an appropriate comparator as an anti-TNF class agent. In addition, adalimumab is dosed every 2 weeks subcutaneously like pateclizumab and the choice of this particular anti-TNF agent reduced the complexity of maintaining the blind by reducing the frequency of injections for patients compared to the use of etanercept. The DAS28-ESR and ACR scores reported for adalimumab in the DMARD-IR population in this study are comparable to historical values for this duration of dosing [25-27].

Pateclizumab had a trend for clinical effects on the signs and symptoms of rheumatoid arthritis after 3 months of treatment in patients with active disease and an inadequate response to DMARDs but the differences from placebo responses were not statistically significant. Pateclizumab had an acceptable safety profile, with no SAEs and no increase in infectious AEs compared to placebo likely related to its limited immunosuppressant effects. Injection-site AEs with pateclizumab were uncommon, typically low grade in severity, and comparable to those with placebo.

The pateclizumab dose regimen evaluated in this study provided expected and sufficient exposure during the treatment period as judged by a rapid and sustained decrease in serum CXCL13 levels at all times out to 85 days. The 40 to 50% inhibition of serum CXCL13 levels in the pateclizumab-treated group in this study confirmed that the exposure of 360 mg (approximately 5 mg/kg based on a median body weight of 72 kg) SC biweekly doses is sufficient to produce maximal downstream pharmacological effect in blocking the LTα pathway. However, although such a pharmacodynamic effect was maximized and similar to the CXCL13 reduction observed in the adalimumab-treated cohort, pateclizumab treatment was not associated with significant clinical benefit.

Despite the reports of elevated levels of LTα and LTβ expression in the synovium of RA patients [5,6,28], as well as the presence of lymphoid aggregates, targeting of the LT pathway with pateclizumab did not have insignificant treatment effects compared to adalimumab. Baminercept, an LTβR fusion protein that only blocks LTα_1_β_2_-LTβR interaction, was evaluated in two clinical trials and found not to be efficacious for the treatment of the signs and symptoms of RA. In a 14-week, dose-ranging phase 2b trial in DMARD-IR patients (N = 391) [29] and in a 14-week, dose-ranging phase 2b trial in TNF-IR patients (N = 114) [9], baminercept did not have significant treatment effects as assessed by ACR50 response rates. In this second study, baminercept did reduce CXCL13 levels by approximately 50% [9], which is similar to our pharmacodynamic results with pateclizumab in DMARD-IR patients.

## Conclusions

Our findings suggest that targeting lymphotoxin signaling, LTα_3_-TNFR interactions and LTα_1_β_2_-LTβR interactions, with pateclizumab on background DMARDs is insufficient to produce a significant reduction in the inflammatory process in RA or to provide superior efficacy over adalimumab, an anti-TNF agent that is commonly used for DMARD-IR RA patients.

## Abbreviations

ADA: adalimumab
AE: adverse event
ALT: alanine aminotransferase
ALTARA: Anti-LTa Rheumatoid Arthritis study
ANCOVA: analysis of covariance
ARC: American College of Rheumatology
AST: aspartate aminotransferase
ATA: anti-therapeutic antibodies
CDR: complementarity-determining region
CRP: C-reactive protein
DAS-ESR(4): 4-variable, 28-joint disease activity score erythrocyte sedimentation rate
DAS28: 3-variable disease activity score in 28 joints
DMARDs: disease-modifying antirheumatic drugs
DMARD-IR: patients who had an inadequate response to DMARDs
ECLA: electrochemiluminescence assay
ELISA: enzyme-linked immunosorbent assay
HAQ-DI: Health Assessment Questionnaire disability index
Ig: immunoglobulin
LOCF: last-observation-carried-forward method
LTα: lymphotoxin alpha
LTβ: lymphotoxin beta
LTα1β2: LT-α complexed with LTβ
LTβR: LTβ receptor
mITT populations: modified intent-to-treat
NCI-CTCAE v3.0: National Cancer Institute common toxicity criteria for adverse events
PK: pharmacokinetic
RA: rheumatoid arthritis
RF: rheumatoid factor
SAE: serious adverse event
SC: subcutaneous
SF: superfamily
SF-36: 36-item Short Form Health Survey
TB: tuberculosis
Th: T helper
TNF: tumor necrosis factor
TNF-α: tumor necrosis factor alpha
TNFR1 and TNFR2: TNF receptors 1 and 2
ULN: upper limit of normal
VAS: visual analog scale
